# Probing Early α‐Synuclein Oligomers: Insights Into Aggregation Pathways of NACore and preNAC Segments Probed by Trapped Ion‐Mobility Mass Spectrometry and Fluorescence Spectroscopy

**DOI:** 10.1002/pmic.70087

**Published:** 2025-12-10

**Authors:** Agathe Depraz Depland, Stephanie Mikromanolis, Iuliia Stroganova, Anouk M. Rijs

**Affiliations:** ^1^ Department of Chemistry and Pharmaceutical Sciences, Division of Bioanalytical Chemistry, Amsterdam Institute of Molecular and Life Sciences Vrije Universiteit Amsterdam Amsterdam the Netherlands; ^2^ Centre for Analytical Chemistry Amsterdam Amsterdam the Netherlands

**Keywords:** early‐stage oligomers, NACore, Parkinson's disease, peptide aggregation, ThT fluorescence, trapped ion‐mobility mass spectrometry

## Abstract

Misfolding and aggregation of α‐Synuclein (α‐Syn) play a central role in Parkinson's disease (PD), with oligomeric intermediates implicated as key toxic species. Here, we investigate the aggregation of two α‐Syn segments, the NACore (_68_GAVVTGVTAVA_78_, WT‐PD1) and the preNAC region (_47_GVVHGVATVA_56_, WT‐PD2), using high‐resolution trapped ion‐mobility mass spectrometry (TIMS‐Qq‐ToF) and Thioflavin T fluorescence spectroscopy. The NACore is the minimal sequence required for α‐Syn aggregation, whereas most mutations affecting the onset of PD appear to be in the preNAC region, therefore modulating aggregation dynamics and toxicity. Our results demonstrate that TIMS‐Qq‐ToF effectively resolves and identifies oligomeric species, revealing distinct aggregation pathways for both peptide segments. Fluorescence assays confirm differences in aggregation kinetics and morphology, highlighting the different oligomer formation pathways observed with ion‐mobility mass spectrometry. Despite the oligomer‐preserving nature of the TIMS itself, ion transmission remains too harsh for the fragile oligomers, leading to fragmentation of fragile non‐covalent assemblies. However, collisional cross‐section (^N2^CCS_TIMS_) values support the presence of large oligomers. This study highlights the potential of TIMS‐Qq‐ToF for mapping α‐Syn aggregation and underscores the need for optimised, soft ion transmission to better preserve fragile transient intermediates, ultimately contributing to a deeper understanding of PD.

**Summary:**

Understanding early‐stage protein aggregation is essential for unravelling the molecular mechanisms of Parkinson's disease, a neurodegenerative disorder that currently lacks effective therapeutic solutions. This study employs high‐resolution Trapped Ion Mobility Mass Spectrometry combined with fluorescence spectroscopy to elucidate the oligomerisation of two α‐synuclein segments, revealing distinct aggregation pathways and associated structural characteristics. This study underscores the value of peptide models in advancing our understanding of protein aggregation behaviour. This multifaceted approach provides detailed structural insights into the unexplored, transient early‐stage oligomers of key α‐Syn segments, contributing to a deeper understanding of aggregation mechanisms and providing valuable insights for therapeutic strategies against Parkinson's disease.

**Abbreviations**
AmOAc: ammonium acetate
CCS: collision cross section
CD: circular dichroism
ESI: electrospray ionisation
IM‐MS: ion mobility mass spectrometry
IR: infrared
NAC: non‐Amyloid Beta component
PD: Parkinson's disease
ThT: Thioflavin T
TIMS: trapped ion mobility spectrometry
WT: wild‐type
α‐Syn: α‐synuclein

## 1 Introduction

Parkinson's disease (PD) is the second most common neurodegenerative disorder and affects approximately 10 million people worldwide [1]. The pathological hallmarks include the degeneration and loss of dopaminergic neurons in the substantia nigra [2], accompanied by the formation of intracellular aggregates composed primarily of α‐synuclein (Lewy bodies) [3, 4]. Typically, proteins have the ability to transform from their soluble, monomeric forms into highly ordered, insoluble aggregates known as amyloid fibrils. Misfolding and aggregation of α‐synuclein (α‐Syn) are central to the pathology of the disease [5, 6]. Recent studies show that oligomeric intermediates rather than the fibrils themselves, possess significant cytotoxicity [7, 8, 9, 10]. Currently, there are no effective therapeutic solutions, highlighting the urgent need for a deeper understanding of the mechanism of protein aggregation at the molecular level. The NACore, a segment of the protein α‐Syn (residues 68 to 78) is the minimal sequence length to represent the aggregation propensity and behaviour of the full‐length protein [11, 12, 13]. Variants and mutations within the pre‐NAC region (residues 47 to 56), while not as toxic, have also been implicated in PD [13, 14, 15].

Fibril formation is inherently stochastic, characterised by the presence of numerous non‐covalently bound, macromolecular intermediates that are transient and present in infinitesimal amounts. While α‐Syn, a protein of broad scientific interest, has been extensively studied [16, 17, 18, 19, 20, 21, 22], these transient oligomers continue to pose significant analytical challenges, leaving many aspects of their aggregation pathways unresolved. A key structural characteristic of oligomerisation is the presence of β‐sheet structural elements, which serve as diagnostic markers to follow the aggregation process. Techniques such as InfraRed spectroscopy (IR) [23, 24, 25, 26, 27], x‐ray diffraction [9, 11, 28], circular dichroism (CD) [29, 30, 31], nuclear magnetic resonance (NMR) [10, 32, 33], or fluorescence spectroscopy [34, 35, 36, 37, 38], allow for the detection of these characteristics. Fluorescence spectroscopy offers versatile approaches for monitoring protein and peptide aggregation, either using a fluorophore present in, or attached to, the protein sequence [39, 40], or using a fluorescent dye such as Thioflavin T (ThT) [34, 41, 42, 43]. ThT is commonly used to follow amyloid formation studies due to its fluorescence upon binding to β‐sheet structures in aggregates. While ThT assays are simple to perform and interpret, their sensitivity to various experimental conditions, such as pH, ionic strength, and temperature, poses a challenge to reproducibility [34, 41, 44]. To confirm the presence of β‐sheet structures indicative of oligomerisation under MS‐based solution conditions and establish a timeline for the aggregation process, standardised conditions compatible with mass spectrometry must be established for each aggregating peptide. Moreover, accurate interpretation of ThT spectra often requires complementary analytical techniques to verify findings at the oligomeric level [45].

In contrast to the spectroscopic methods discussed above, which provide population‐averaged data, mass spectrometry (MS) enables the separation of individual oligomers. MS offers a unique advantage in identifying these species, including minor entities within complex mixtures. Electrospray ionisation coupled to mass spectrometry (ESI‐MS), and especially nano spray ESI (nano‐ESI), is particularly advantageous for preserving non‐covalent interactions from the solution to the gas phase [46, 47, 48, 49]. Moreover, the rapid data acquisition of MS allows for real‐time monitoring of the formation and disappearance of oligomers along the aggregation pathways [30, 45]. Ion mobility mass spectrometry (IM‐MS) extends the capabilities of traditional MS by incorporating ion mobility analysis, allowing for the separation of ions based not only on their mass‐to‐charge ratio (*m*/*z*) but also on their shape and size. This additional dimension of analysis provides valuable insight into the conformational landscape of protein and peptide aggregates, and more importantly it allows the distinction between different oligomeric species within the same *m*/*z* channel [30, 50, 51, 52, 53, 54, 55]. Trapped ion mobility mass spectrometry (TIMS) provides high‐resolution mobility separation and has been reported to potentially preserve the intact structure of non‐covalent peptide assemblies when operated under so‐called ′soft conditions′ [56, 57, 58, 59, 60]. IM‐MS has proven to be highly effective in probing the dynamics of oligomer formation along the aggregation pathway and has been useful in elucidating the mechanisms by which specific conformations may contribute to neurotoxicity. For example, Laos et al. have studied the aggregating peptide segment of the TDP‐43 protein using a drift‐tube ion mobility coupled to mass spectrometry, confirming the ability to follow aggregation pathways using IM‐MS [30]. However, challenges remain, particularly with respect to preserving non‐covalent interactions, especially when using commercial instruments, although they benefit from higher resolving power and the accurate representation of conformational states in large assemblies during analysis [51, 56, 61, 62].

In this study, we aim to demonstrate the capabilities of the high‐resolution TIMS‐Qq‐ToF classic spectrometer to monitor and separate early‐stage aggregates over time. In previous studies, the softness of the TIMS mass spectrometer has been improved, resulting in optimised ion manipulations [56, 58, 63] Here, we apply this advanced method to analyse the oligomerisation of two α‐Syn segments, the NACore and the preNAC region, over time. In addition, fluorescence spectroscopy based on the ThT dye was used to confirm the presence of β‐sheet structures indicative of oligomerisation, to establish a timeline for the aggregation process, and characterise the morphologies of the resulting aggregates. Through this combined approach, we aim to map the aggregation pathways of α‐Syn segments, providing insights into the molecular mechanisms underlying Parkinson's disease.

## 2 Material and Methods

### 2.1 Sample Preparation

Both wild‐type segments 68–78 (WT‐PD1) and 47–56 (WT‐PD2) of the α‐Syn protein, with sequences _68_GAVVTGVTAVA_78_ and _47_GVVHGVATVA_56_, and molecular weights of 943.53 and 908.51 Da, respectively, were purchased from Biomatik and used without any further purification (>95%). First, a process of aliquoting was performed, which is described in detail elsewhere [45, 58]. The peptide stock solution (100 µM) was prepared in a 10 mM ammonium acetate solution with pH 7.3–7.45 (AmOAc), using a 5 M AmOAc in water and an ammonia solution 25% (Sigma–Aldrich) to adjust the pH. The peptide: AmOAc solution was briefly vortexed (2–3 s) to ensure proper dissolution of the sample and homogenisation. The stock solution was then sonicated for 20 min to initiate the aggregation process. Subsequently, the sample was incubated at 37°C until the end of the experiment.

### 2.2 Trapped Ion Mobility Mass Spectrometry Using nanoESI

The ion mobility–mass spectrometry (IM‐MS) experiments were conducted using a first‐generation classic TIMS‐Qq‐ToF mass spectrometer (Bruker Daltonics) [61, 64, 65]. The spectrometer was equipped with the 3D‐printed static nano‐ESI source designed by Götze et al. [47]. The use of nanoESI is essential for probing oligomer formation overtime [58, 59]. The capillaries were pulled using the P‐1000 (Sutter instrument) and gold‐coated with a sputter coater. The tips of the nano‐emitters were cut with tweezers under an optical microscope.

The ion mobility was measured from 0.7 to 1.7 1/K_0_ (or Vm^−1^s^−2^) and mass spectra were recorded over the range from 0 to 3000 *m*/*z*. The 100 µM aggregating sample was diluted to 5 µM and transferred into the coated glass capillary for infusion in the MS operating in positive mode. The capillary voltage was varied between 600 and 1400 V to obtain the best signal while preserving the fragile assemblies. Quadrupole filtering after mobility separation was set at 944.5 *m*/*z* (±5 *m*/*z*) for WT‐PD1 and at 909.5 *m*/*z* (±5 *m/z*) for WT‐PD2. Further instrumental parameters are reported in Table S1. The instrument was calibrated on the tunnel‐in pressure used during the measurements for mass and mobilities using the Agilent ESI tune mix prior to all analyses [52]. External calibration was performed in the DataAnalysis software after each acquisition using the Tuning Mix calibration file measured under identical operating settings.

### 2.3 Fluorescence Assays

A stock solution of 100 µM peptide solution, with a ratio of 1:1, peptide dye concentration was prepared following the protocol described by Laos et al. [30, 42]. The peptide‐buffer‐ThT solution was vortexed briefly (2–3s) and subsequently sonicated for 20 min. At this point, 100 µL of the solution was pipetted into two wells of a 384‐well plate (black plate with clear and flat bottom, Greiner), and covered with a breathable sealing tape. Fluorescence was monitored using the ThermoScientific Varioskan LUX Multimode Microplate Reader (Fisher Scientific), for 16 consecutive hours for both WT‐PD1 and WT‐PD2, under quiescent conditions. The excitation and emission wavelengths for ThT were set at 445 and 490 nm, respectively. The plate reader was used with the incubator at 37°C, and measurements were taken every 20 min with a measurement time of 100 ms using the top optics. Additional plate reader settings are reported in Table S2.

### 2.4 Fluorescence Microscopy

Fluorescence microscopy was performed using an Operetta CLS fluorescence microscope at 40× magnification on samples prepared as previously described for the ThT assays. After preparation of the stock solution, 20 µL was pipetted into one well of the CellCarrier 384 Ultra well plates (Perkin Elmer) as a first time point measurement. The solution was then sonicated, after which two additional wells were filled with 20 µL. The microscope spatially measured the fluorescence in different areas of the well, see Figure S1. For each area, an image was taken every 8 µm across the volume of the well. The temperature was set at 37°C. Measurements were typically taken over several hours, with intervals of 15 min for the first 4 h, and then every hour up to 55 h.

## 3 Results and Discussion

We investigated the aggregation process of two different segments of α‐Syn, _68_GAVVTGVTAVA_78_ (WT‐PD1) and _47_GVVHGVATVA_56_ (WT‐PD2), using a combination of analytical techniques, see Figure S2A,B for their chemical structures. Both peptide segments were prepared and analysed in the same manner, whether the aggregation was measured with IM‐MS or fluorescence spectroscopy, as described in the method section. The method used for the TIMS experiments was developed to ensure softness of the parameters and thus maximum preservation of the fragile peptide assemblies formed during the aggregation process [58].

### 3.1 Monitoring the Aggregation Pathways of WT‐PD1

#### 3.1.1 ThT Binding Fluorescence of WT‐PD1

**FIGURE 1:**
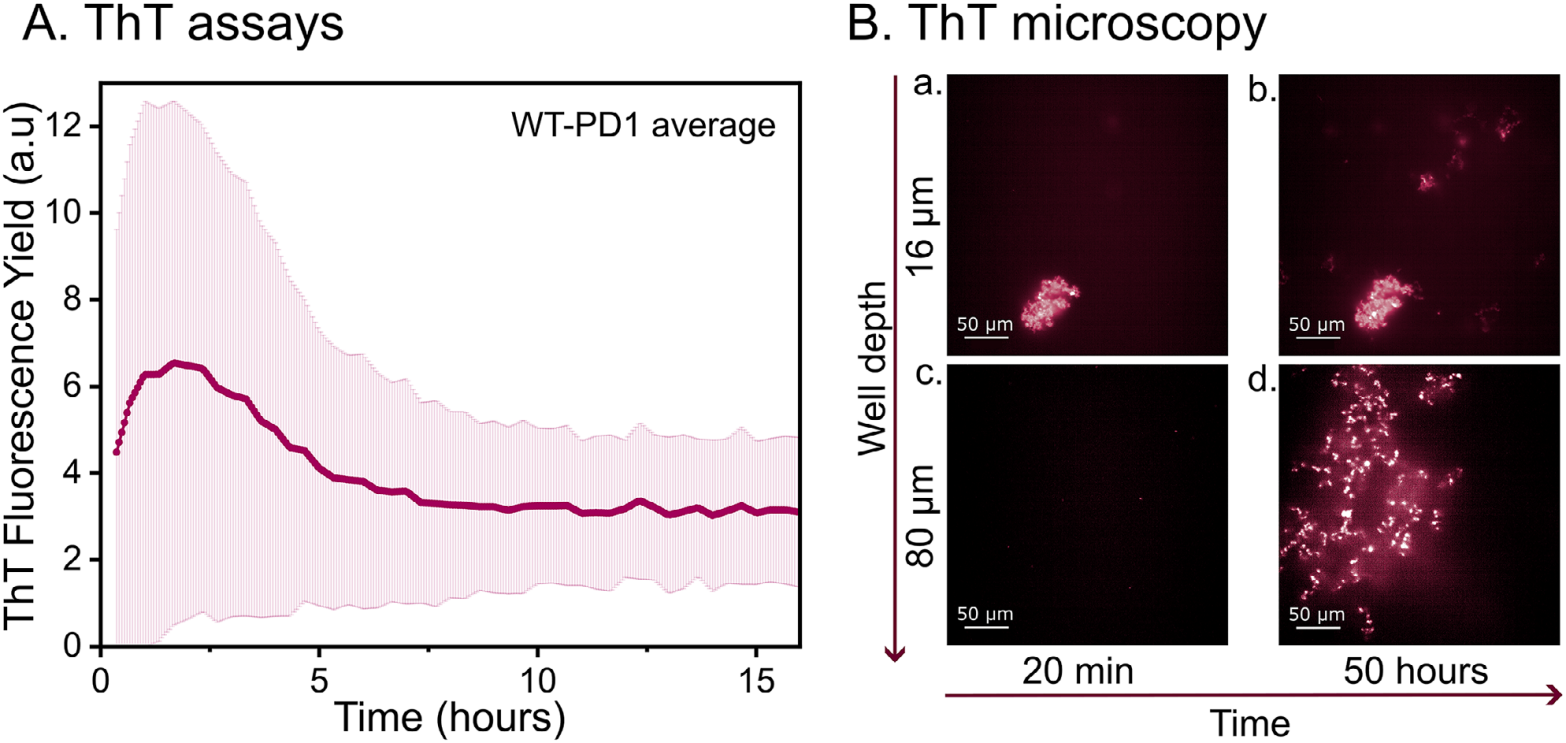
WT‐PD1 peptide from the NAC region of α‐Synuclein measured with: (A) Fluorescence assay with ThT dye starting after 20 min of sonication. Five averaged replicated measurements plotted with the corresponding standard deviation plotted as error bars. (B) ThT fluorescence microscopy images after (a) and (c) 20 min and (b) and (d) 50 h, at well depth of 16 and 80 µm (depth from bottom to top). The scale in the picture represents 50 µm.

The averaged fluorescence yield of five identical experiments is presented in Figure 1A, where we can observe the aggregation from the growth phase to the plateau/saturation phase. This corresponds to the transition of the peptides from small soluble aggregates to organised β‐sheet structures, which typically occurs within the first 10 h after solvation of the analytes. A 20‐min sonication of the sample is performed upon solvation to initiate the aggregation process, which otherwise does not occur within the 16‐h time frame of the experiment, as observed in Figure S3. After 20 min of sonication, the fluorescence yield rapidly increases to its maximum. This is attributed to the growth phase of the aggregation process. Note that the corresponding fluorescence intensity can vary depending on the experimental workflow. The plateau phase is defined as saturation of the ThT‐peptides complexes formed, which is here determined as the point where the fluorescence reaches its maximum intensity, at approximately 2.5 h (i.e., the end of the growth phase). Following the growth phase, the plateau phase is observed as a declining signal, which stabilises after approximately 10 h. This decrease is most likely due to self‐quenching of the ThT, which happens typically when high concentrations of ThT are used (>50 µM) and the oligomers‐ThT complexes become more compact during their growth [30, 36, 42, 43, 66]. Since the decrease is continuing for approximately 6 h, we can assume that the aggregation is still ongoing. However, we expect the sample to be dominated by large structures such as fibrils rather than the smaller, highly fluorescent β‐sheet oligomers. Consequently, the quenching of the fluorescent signal is dominant over the increase in fluorescence signal. Eventually, the signal stabilises, suggesting that the aggregation changes are too weak to be detected with this instrument.

Figure 1B represents the initial (after 20 min of sonication) and final structure morphologies (at 55 h) observed by ThT fluorescence microscopy at two different locations in the well volume. Images from these well areas prior to sonication and for a selection of time intervals of the experiment are provided in Figures S4A and S5, respectively. These images show that no β‐sheets (e.g., no fluorescence signal) are detected prior to the sonication step and that the aggregates reach a stable structure between 10 and 30 h after sample preparation. This time evolution observed with microscopy shows that the aggregation does not stop completely when the fluorescence signal reaches a plateau (Figure 1A). It is possible that the plate reader is simply not sensitive enough to monitor weaker changes in fluorescence intensity.

Two types of structures are observed in the image presented in Figure 1B. First, a small, compact structure that appears at the bottom of the well (Figure 1B(a),(b)), which remains the same over time. This feature results from sonication, as it appears after 20 min but is absent in pre‐sonication images (Figure S4A). Additionally, small, faint structures emerge in the top right at 55 h (Figure 1B(b)), corresponding to the edge of a larger structure extending upward. A broader (>200 µm), less dense structure develops over time near the vertical centre (Figure 3B(c),(d)), spanning more than half the well area.

Both structures contribute to the total fluorescence yield in the ThT assay, which averages signals across the well. This explains the fluorescence detected from the start (Figure 1A), as sonication appears to initiate aggregation and form large fluorescent complexes. These results confirm NACore segment aggregation under our experimental MS‐compatible conditions, as fluorescence detection indicates β‐sheet presence. Additionally, they establish a timeline for small, soluble oligomers in IM‐MS, expected between the lag phase and plateau, approximately 2.5 h post‐dissolution.

#### 3.1.2 Ion Mobility‐Mass Spectrometry of WT‐PD1 Over Time

**FIGURE 2:**
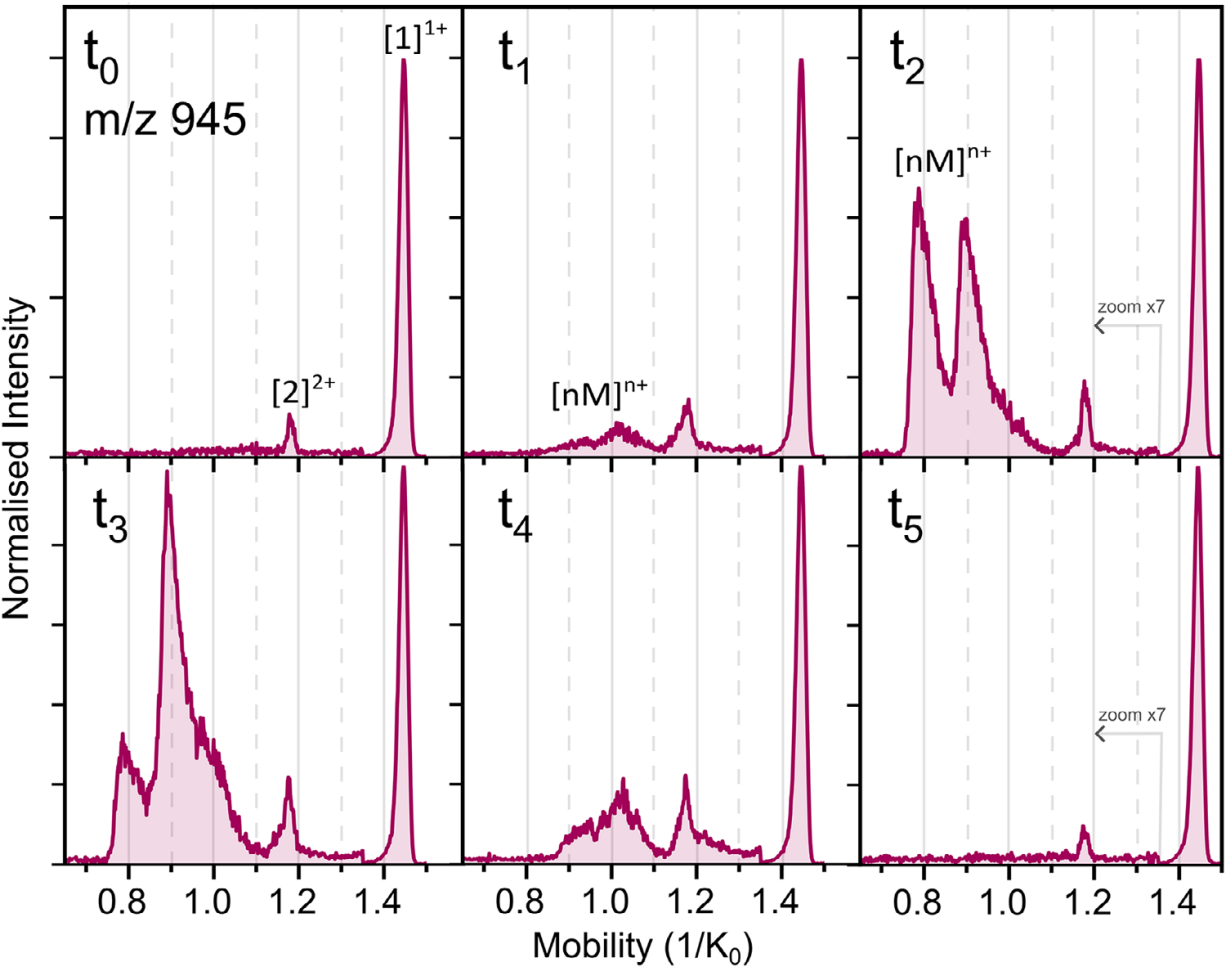
Extracted ion‐mobility spectra of WT‐PD1 from NAC region of α‐Synuclein, 944.5 *m*/*z* at dilution times from *t*_0_–before sonication–to *t*_1_–after sonication–to *t*_5_ the latest dilution. Intensities are normalised to the intensity of the [1]^1+^ of *t*_0_, and the intensities of the mobility values under 1.35 1/K_0_ are multiplied by 7 for all time points equally.

The oligomeric assembly of WT‐PD1 was examined by IM‐MS over a period of several hours. Each new time point corresponds to the time when a new dilution was made from the stock solution. Time points were measured from the dissolution of the peptide in ammonium acetate solution (*t*_0_), and then about every 20 min. In Figure 2, the quadrupole filtered *m*/*z* 944.5 mobility spectra of six selected consecutive time points are plotted in the mobility range from 0.7 to 1.5 1/K_0_, with the earliest time t_0_ (±5 min) and the latest time t_5_ (>2 h). WT‐PD1 displays a linear type of oligomerisation, meaning a linear increase of both mass and charge of the ions. This results in [nM]^nz+^ type of oligomers (*n* = number of monomeric units, *M* = protonated monomeric mass, and z = charge), where each peptide assembly formed is measured at the same *m*/*z* channel.

Two peaks are observed at *t*_0_, a very intense main peak at 1.45 1/K_0_, corresponding to the singly charged monomer ([1]^1+^), and a second peak at 1.18 1/K_0_, about 40 times less intense, that corresponds to the doubly charged dimer ([2]^2+^). Both the [1]^1+^ and [2]^2+^ are present in all mobility spectra over time with a stable intensity ratio. After sonication of the stock solution, a new dilution is made (*t*_1_) resulting in two additional broad peaks in the mobilogram at 0.94 and 1.02 1/K_0_. The isotope distributions extracted from these mobility peaks do not allow for unambiguous identification of the oligomers (see Figures S6 and S7).

**FIGURE 3:**
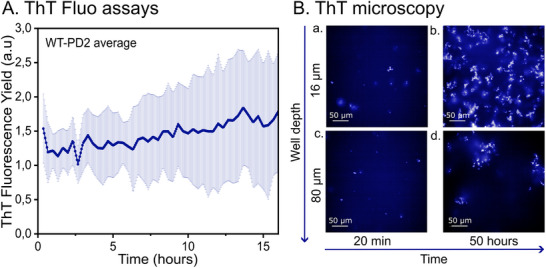
WT‐PD2 peptide from the pre‐NAC region of α‐Synuclein measured with: (A) Fluorescence assay with ThT dye starting after 20 min of sonication. Five averaged replicated measurements plotted with the corresponding standard deviation error bars, and (B) ThT fluorescence microscopy pictures after (a) and (c) 20 min and (b) and (d) 50 h, at well depth of 16 and 80 µm (depth from bottom to top). The scale in the picture represents 50 µm.

The presence of higher order oligomers is therefore assessed by the appearance of lower reduced mobility values peaks, corresponding to the growth in mass and charge of the ions present under this filtered *m*/*z* ratio. Between *t*_1_ and *t*_3_, the mobilograms show an increase in signal intensity at lower reduced ion mobility values. At *t*_2_, two distinct broad peaks appear at 0.90 and 0.79 1/K_0_ with an intensity three times higher than the dimer peak, thereby masking the previously observed peaks (at *t*_1_) in this mobility region. At *t*_3_, the most intense oligomeric presence is observed, after which the mobility peaks below 1.13 1/K_0_ decrease again in intensity until they fully disappear, leaving only the monomer and dimer peaks. The mobility spectra, as well as the mass spectra (Figure 3A,C) of the last time point (*t*_5_) are identical to the first time point (*t*_0_), showing no sign of oligomers.

The overall trend observed with IM‐MS involves a preliminary phase in which no oligomers are detected. This is indicative of the initial stages of the lag phase. It is rather short‐lived under our experimental conditions (maximum 20 min), as the sonication accelerates the aggregation process. In the absence of sonication, this lag phase would occur over longer time frames, as observed from the fluorescence curves with and without sonication in Figure S3. At a subsequent time point, an increase in the size of the oligomers is observed, coinciding with an increase in the lower reduced ion mobility values. During this growth phase, peptide assemblies are detected by IM‐MS until they reach a size at which the oligomers are no longer soluble and therefore cannot be ionised. From that point (*t*_3_) and extending up to *t*_5_, there is a decline in the soluble aggregate abundance until their total disappearance in the mobility spectra. The data presented in Figure 2 offers the most comprehensive picture of the temporal progression of events observed for WT‐PD1 with IM‐MS. Given the stochastic nature of the aggregation process, it is not uncommon that only part of the process can be observed within a single IM‐MS experiment, as shown in Figure S8.

The presence of soluble oligomers can also be observed in the MS, although not to the same extent. The *t*_0_, *t*_2,_ and *t*_5_ MS corresponding to the mobilograms from Figure 2 are plotted in Figure S7, showing an increase of the baseline at the time point where oligomers are present (*t*_2_). This baseline increase is not observed at *t*_0_ before aggregation or at *t*_5_ after aggregation, when oligomers are not observed in the mobility spectra. This broad baseline increase is characteristic of oligomeric growth; however, it is relatively low when measured with the TIMS‐Qq‐ToF [30]. Here, the isotopic distribution does not allow to assert the presence of oligomers.

#### 3.1.3 Monitoring the Aggregation Pathways of WT‐PD2

The second segment (_47_GVVHGVATVA_56_), which can be found in the pre‐NAC region of α‐Syn, has a very similar amino acid sequence to the NAC segment previously discussed. The pre‐NAC WT‐PD2 sequence also contains a histidine residue at the 50^th^ position, in addition to glycine, valine, threonine, and alanine, see Figure S2B.

#### 3.1.4 ThT Binding Fluorescence of WT‐PD2

Figure 3A presents the averaged fluorescence yield from five ThT binding assays of WT‐PD2. Aggregation begins independently of sonication, although this step was included for consistency with the study of WT‐PD1. ThT fluorescence is detected from the beginning, decreases briefly (∼1 h), and then gradually increases over 16 h. The overall signal is low (max 2.8 a.u., avg. 1.5 a.u.) compared to WT‐PD1 (max 12 a.u., avg. 5 a.u.). This suggests that structures to which the fluorescent ThT can bind are formed immediately upon solvation, as confirmed by the pre‐sonication image obtained using fluorescence microscopy (Figure S4B). Sonication may disrupt these structures and their binding to ThT, potentially explaining the initial decrease. Subsequently, after >1h, a slow fluorescence growth is observed, indicating possible multiple aggregation phases. The low fluorescence intensity suggests the presence of either a limited amount of β‐sheets or structures exhibiting a low binding affinity with the ThT, indicating an atypical aggregate structure, at least different from WT‐PD1 [67, 68]. This hypothesis is supported by the features observed with fluorescence microscopy.

Pre‐sonication images (Figure S4B) show large, amorphous fluorescent structures with no temporal variation, which disappear after sonication. Post‐sonication images (Figure 3B(a),(c)) reveal small (<10 µm) fluorescent dots scattered throughout the well. At 16 µm depth (Figure 3B(a),(b)), small dense structures form at the bottom of the well that increase in number, but not size, over 3 h (Figure S9A). At 80 µm depth (Figure 3B(c),(d)), larger (∼100 µm) WT‐PD1‐like structures appear and grow for at least 15 h (Figure S9B). These distinct pathways are visible by fluorescence microscopy but are averaged in fluorescence intensity assays, obscuring differences and preventing kinetic analysis. Early oligomers could be detected by IM‐MS from sonication up to 15 h.

#### 3.1.5 Ion Mobility‐Mass Spectrometry of WT‐PD2

As previously discussed for WT‐PD1, the oligomeric assembly of WT‐PD2 was studied by IM‐MS over a time period of several hours. Time points were measured from the dissolution of the peptide in an AmOAc solution (*t*_0_), and subsequently every 20 min on average. As for WT‐PD1, WT‐PD2 displays a linear oligomerisation resulting in [nM]^nz+^ type of oligomers, where each peptide assembly formed is measured in the same *m*/*z* channel. The quadrupole‐filtered *m*/*z* 909.5 mass spectra at four different consecutive time points show the presence of aggregates. As previously mentioned, these are not sufficient to characterise the aggregation process, as shown in Figure S11B,D.

**FIGURE 4:**
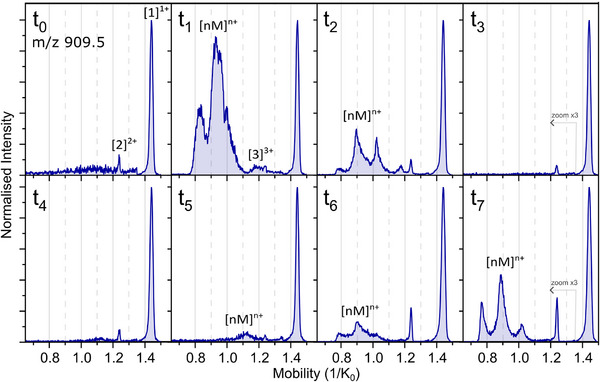
Extracted ion‐mobility spectra of WT‐PD2 from pre‐NAC region of α‐Synuclein, 909.5 *m*/*z* at dilution times from *t*_0_–before sonication–to *t*_1_–after sonication–to *t*_7_ the latest dilution. Intensities are normalised to the intensity of the [1]^1+^ of *t*_0_, and the intensities of the mobility values under 1.35 1/K_0_ are multiplied by 3, for all time points equally.

Figure 4 presents the mobility spectra of a selection of eight consecutive time points in the mobility range from 0.7 to 1.5 1/K_0_, with the earliest time *t*_0_ (±5 min), the end of the first phase *t*_3_ (±1 h), and the latest time *t*_7_ (about 2 h), showing two subsequent phases of oligomeric growth. In the first phase from *t*_0_ to *t*_3_, as depicted in Figure 4, an intense and rapid increase and then decrease in the signal of the lower mobility values is observed.

The first spectrum (*t*_0_) shows only two peaks at 1.44 and 1.24 1/K_0_, corresponding to the monomer singly charged ([1]^1+^) and the dimer doubly charged ([2]^2+^), respectively. An elevated baseline below 1.24 1/K_0_ is observed, which is analogous to our findings reported for WT‐PD1. From the second measurement *t*_1_, immediately after the sample was sonicated, the [2]^2+^ peak at 1.24 1/K_0_ almost merges into a broad feature extending from 1.24 to 1.14 1/K_0_. This feature shows the weak presence of a [3]^3+^ isotopic distribution, shown in Figure S10, among a larger contribution of [1]^1+^ and [2]^2+^ ions. This observation indicates strong fragmentation of the [3]^3+^ ions (into [1]^1+^ and [2]^2+^) after the TIMS mobility cell and prior to the arrival of the ions at the ToF analyser [45, 56, 58, 61].

Additionally, we observe an intense signal between 0.8 and 1.1 1/K_0_ with three possible maxima at 0.99, 0.94 and 0.83 1/K_0_. These peaks are not baseline separated and appear broader than the previously observed monomer and dimer peaks. Similar to what was observed for WT‐PD1, the isotopic distributions extracted from the mobility values below the trimer peak, here 1.14 1/K_0_, typically do not allow the unambiguous identification of the ions thereafter labelled [nM]^n+^.

The extracted isotopic distributions in the mass spectra from each mobility peak observed in Figure 4 can be found in Figure S11. At *t*_2_ a similar distribution to *t*_1_ is observed, but the peaks appear narrower and more pronounced. At *t*_3_, the higher‐order oligomeric peaks have disappeared, leaving only [1]^1+^ and [2]^2+^ ions. *t*_4_ marks the beginning of a second aggregating phase. From this point until *t*_7_, a new, slower growth of oligomers can be seen, showing the presence of the same ions as observed at *t*_2_ and *t*_3_, but with different ratios, and with increasing intensity over time. The [3]^3+^ was not detected in these ion mobility spectra. At the last time point *t*_7_, the oligomers still persist.

What we observed with the fluorescence assays is in agreement with the IM‐MS results. A weak increase of fluorescent intensity was observed after the sonication (Figure 3A), coinciding with the significant signal of oligomers (Figure 4 *t*_1_ and *t*_2_). Subsequently, the oligomer intensity in IM‐MS and in the fluorescence assay are decreasing for about an hour. However, after about an hour, the oligomer population in IM‐MS is increasing again, which can indicate the start of the second slower self‐assembly phase, as was observed with fluorescence assay measurements over the remaining 15 h of measurement. Fluorescence microscopy allows for the observation of the morphologies of the structures created during the self‐assembly, showing that two types of aggregates have been formed. While observing obvious signs of aggregation both in IM‐MS and in fluorescence, the overall fluorescence intensity signal is low, from both the assays and the microscopy experiments. This indicates that ThT is not binding as strongly to WT‐PD2 assemblies, as compared to WT‐PD1, suggesting that the aggregates formed do not fully consist of β‐sheets. Due to the inherent variability in these experiments, the data in Figure 4 are the most representative of WT‐PD2 self‐assembly, while some replicates of the experiment captured only part of the process, as shown in Figure S12.

#### 3.1.6 Using Mobility Measurements to Determine CCS Values

**FIGURE 5:**
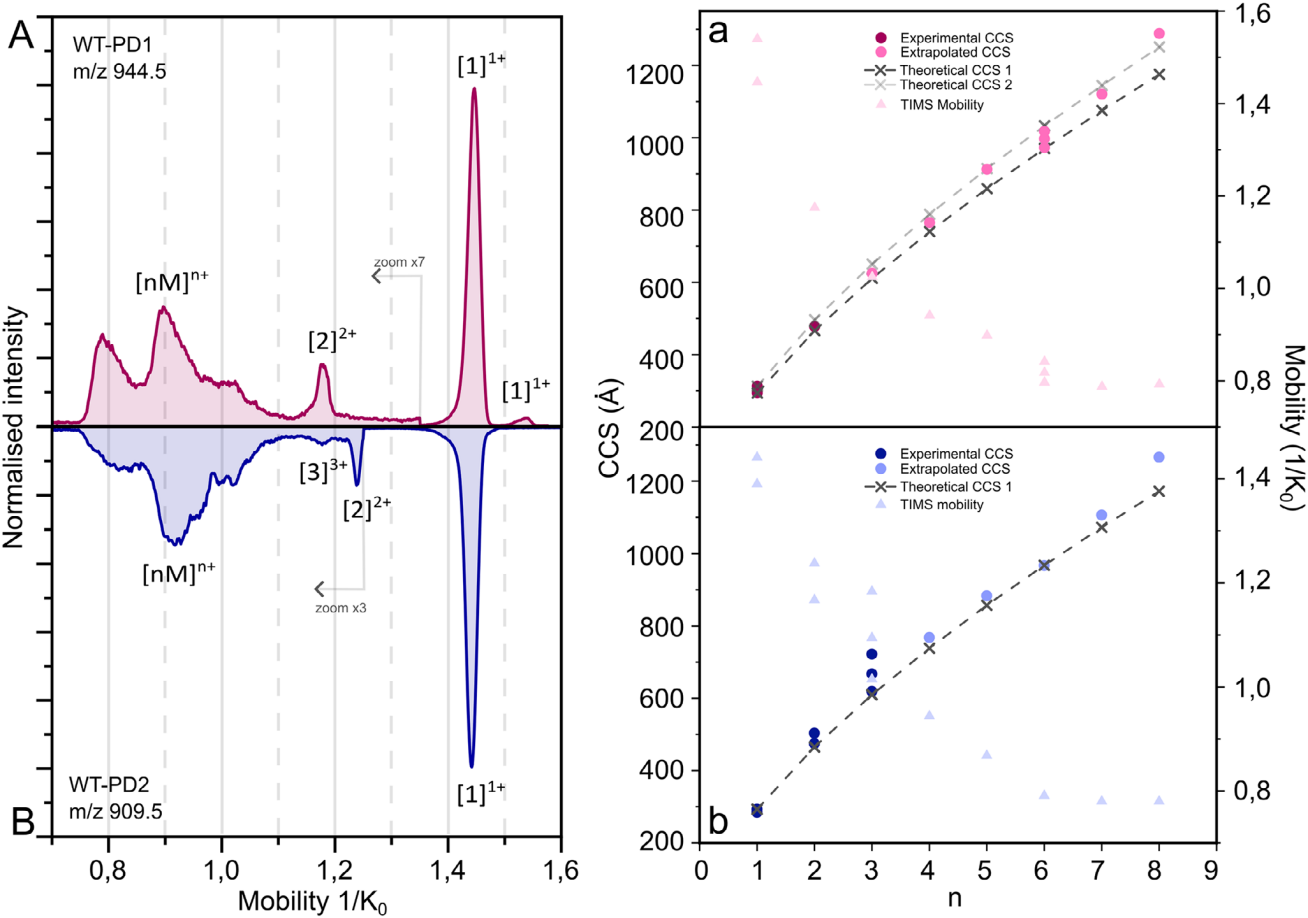
Averaged normalised mobility spectra of A. all mobility spectra of WT‐PD1 from Figure 2, with intensities multiplied by 7 below 1.35 1/K_0_ and B. all mobility spectra of WT‐PD2 from Figure 4, with intensities multiplied by 3 under 1.25 1/K_0_. a and b show, on the left axis plotted in grey with a dotted line the theoretical ^TIMS^CCS_N2_ calculated from the isotropic curve using the [1]^1+^ value determined from the mobility spectra and calculated according to this formula: CCS_n_ = *n* ^2/3^ x CCS_1_, in A and B. In dark pink and blue, the experimental values from which the identity was confirmed using isotopic distribution calculated to ^TIMS^CCS_N2_ and in light pink and blue the extrapolated values, equal to or larger than the ones indicated in the plot, from which the isotopic distribution did not allow for the identification of the ions. On the right axis, light pink and blue triangles, the corresponding mobility values.

The combination of techniques presented in the previous sections allowed the qualitative characterisation of the aggregation of WT‐PD1 and WT‐PD2 segments. We observed that their organisation behaviour is quite different, first in the evolution over time of the species present and detected with IM‐MS, and second in the fluorescence yields obtained, showing that the possible formation of β‐sheets and their binding with the ThT dye are different. Finally, the microscopy images confirm that quite different structures are being formed, as the shape, the position in the well and the density are different. A comparison of these data reveals that, under our experimental conditions, WT‐PD1 has a linear aggregation pathway, resulting in the formation of broad fibril‐like structures. In contrast, the aggregation process of WT‐PD2 gives rise to the formation of different structures that are both smaller and denser than the β‐sheet oligomers observed for WT‐PD1. The averages of all time points measured with IM‐MS for both the WT‐PD1 and WT‐PD2 peptides have been plotted in a composite spectrum representing all possible species present throughout the aggregation process, see Figure 5AB. When examining the peaks present in the mobilograms, at first sight, both peptides show similar features, see Figure 5A.

To better understand the species represented by the mobility peaks, ^TIMS^CCS_N2_ values were calculated using the reversed reduced mobility values extracted from the mobilograms (Figure 5B). The isotopic distribution obtained for selected ions allowed the assignment of CCS values with confidence, including the singly charged monomers and the doubly charged dimers ([1]^1+^ and [2]^2+^) for both peptides, and additionally the triply charged trimers ([3]^3+^) of WT‐PD2. These values are represented in darker colours in Figure 5B(a,pink),(b,blue) for WT‐PD1 and WT‐PD2, respectively. From the ^TIMS^CCS_N2_ values of the singly charged monomers, isotropic curves were traced, as performed by Bowers et al. in previous studies [69], shown in grey in Figure 5B(a),(b), representing the isotropic growth of the monomeric units. Since the lower mobility peaks do not show a clear isotopic distribution corresponding to higher charge states, nor do they show clear fragments allowing identification of the ions [45], as can be observed in Figures S6 and S10, the possible ^TIMS^CCS_N2_ values were extrapolated. These extrapolated ^TIMS^CCS_N2_ values are shown in light colours in Figure 5B(a),(b).

For this, the ^TIMS^CCS_N2_ from each mobility have been determined using multiple charge states (*z*) in DataAnalysis (Bruker), typically between 3 and 12 positive charges with corresponding monomeric unit numbers (*n*). Then the value closest to the isotropic curve [50] was retained. This allows us to assess that the ^TIMS^CCS_N2_ of the ions, from each peak in the mobility spectra, has to be equal to or larger than this retained value. This indicates that the presence of oligomers of at least 8 units detected in the mobility spectra is plausible.

The low mobility peaks are relatively broad compared to the higher mobility, smaller ions. For larger oligomeric ions, the CCS would deviate further from the isotropic curve, as would be expected since their arrival time distribution broadens with their size and conformational diversity. Additionally, the possibility that oligomers with similar mobility values are not resolved by the instrument could also contribute to the broadening.

## 4 Concluding Remarks

The temporal evolution of the oligomerisation of NACore and preNAC peptide segments of α‐Syn has been probed using a multifaceted approach based on ion mobility mass spectrometry and fluorescence spectroscopy. The high resolving power of the TIMS‐Qq‐ToF instrument allows for a good separation and identification of the oligomers, and the observation of the different pathways followed by the soluble peptide segments. However, these studies could benefit from the softer ion transmission as the ions pass through the mass spectrometer, while maintaining optimal sensitivity and resolving power. The TIMS‐Qq‐ToF classic has a relatively soft ion mobility separation, preserving many oligomers during mobility separation. However, the transmission from the mobility cell to the ToF analyser still induces notable oligomer fragmentation. Fragmentation due to ion heating, observed in the mass spectra, complicates the unambiguous identification of larger peptide aggregates.

Strictly analysing the mobility peaks observed by IM‐MS, WT‐PD1 and WT‐PD2 appear to form similar types of aggregates. However, the ThT binding fluorescence assays and the corresponding fluorescence microscopy images clearly demonstrate that both peptide segments follow distinct aggregation pathways under similar experimental conditions, leading to different structural morphologies at later stages of aggregation. The extrapolated ^TIMS^CCS_N2_ values, found close to the isotropic curve, align well with the presence of larger oligomers of increasing *m*/*z* observed by IM‐MS. However, this is insufficient to unambiguously confirm the identity of the ions. An additional dimension of measurement, such as oligomer‐selective infrared action spectroscopy, would be essential to characterise the secondary structures formed and to determine the similarities or differences that arise in both aggregation pathways.

## Funding

This work is supported by the funding from the research program VICI with project number VI.C.192.024 and Aspasia (015.015.009) from the Dutch Research Council (NWO) awarded to A.M.R.

## Conflicts of Interest

The authors declare no conflicts of interest.

## Supporting information




**Supporting File**: pmic70087‐sup‐0001‐SuppMat.pdf.

## Data Availability

The data are available via https://doi.org/10.48338/VU01‐6MXABZ.
