# Supplementary material for: Probing Early α‐Synuclein Oligomers: Insights Into Aggregation Pathways of NACore and preNAC Segments Probed by Trapped Ion‐Mobility Mass Spectrometry and Fluorescence Spectroscopy
Source: Proteomics. 2025 Dec 10;26(4):49–59. doi: 10.1002/pmic.70087 (PMC13048457; doi:10.1002/pmic.70087)
Supplement: Supplementary file 1 — Supporting File: pmic70087‐sup‐0001‐SuppMat.pdf. [file PMIC-26--s001.pdf]

Supplementary Information

Mapping early α-Synuclein oligomers: Insights into aggregation pathways of NACore and preNAC segments probed by Trapped Ion-Mobility Mass Spectrometry and ThT fluorescence

Agathe Depraz Depland[1,2], Stephanie Mikromanolis[1], Iuliia Stroganova[1] and Anouk M. Rijs[1,2]

1. Division of Bioanalytical Chemistry, Amsterdam Institute of Molecular and Life Sciences,
Vrije Universiteit Amsterdam, De Boelelaan 1105, 1081 HV Amsterdam, the Netherlands
2. Centre for Analytical Chemistry Amsterdam

Table of Content

**Table S1**	TIMS instrumental parameters for WT-PD1 experiments	*1*
**Table S2**	Plate reader instrumental parameters for WT-PD1 experiments	*1*
**Figure S1**	Fluorescence microscope experimental setting scheme	*2*
**Figure S2**	WT-PD1 and WT-PD2 chemical structures	*2*
**Figure S3**	Effect of sonication and ionic strength of AA buffer on ThT fluorescence binding assay of WT-PD1	*3*
**Figure S4**	Fluorescence microscopy images recorded without sonication at 5 minutes and 55 hours after sample preparation for WT-PD1 and WT-PD2	*4*
**Figure S5**	Fluorescence microscopy images of WT-PD1 over time for two different well depths	*5*
**Figure S6**	Extracted MS spectra of WT-PD1 from each mobility peak present over time and corresponding MS zoomed on isotopic distribution	*6*
**Figure S7**	Zoomed MS spectra of WT-PD1 at time points pre, peri and post-aggregation	*7*
**Figure S8**	Replicates of the mobility over time measurements of the selected 944.5 *m/z* from WT-PD1	*8*
**Figure S9**	Fluorescence microscopy images of WT-PD2 over time for two different well depths	*9*
**Figure S10**	Extracted MS spectra of WT-PD2 from each mobility peak present over time and corresponding MS zoomed on isotopic distribution	*10*
**Figure S11**	Zoomed MS spectra of WT-PD2 at time points pre, peri and post-aggregation	*11*
**Figure S12**	Replicates of the mobility over time measurement of the selected 909.5 *m/z* from WT-PD2	*12*

Table S1 – TIMS instrumental parameters for WT-PD1 experiments

	Parameters	**WT-PD1**
**Source**		Nano-ESI
	Temperature (°C)	80
	Capillary voltage (V)	Variable (600-1400)
	Dry gas (L/min)	1.5
	Nebulizer (Bar)	0 to 0.1
**MS**		
	Funnel 2 RF (Vpp)	100
	Multipole RF (Vpp)	50
	Ion energy (eV)	10
	Collision energy (eV)	1
	Collision Cell RF (Vpp)	1300
	IMS Collision cell In (V)	200
**TIMS**		
	Δ6 (V)	2
	Δ1 (V)	0
	Δ2 (V)	0
	Δ3 (V)	20
	Δ4 (V)	40
	Δ5 (V)	0
	Accumulation time	2
	Ramp time (ms)	100
	Funnel 1 RF (Vpp)	250
	Tunnel Out Vacuum (mBar)	0.95
	Tunnel In Vacuum (mBar)	2.40

Table S1- Recapitulative table of the parameters for both the standard and the optimised method. °C corresponds to temperature in degree Celsius, Vpp corresponds to Volt peak to peak, eV corresponds to electron Volt, L/min corresponds to litre per minute, ms corresponds to milliseconds, mBar corresponds to milliBar, and V corresponds to Volts.

Table S2 – Plate reader instrumental parameters for WT-PD1 experiments

**Instrument information**	
Set temperature	37°C
Optical response compensation	Yes
Incubator	Yes
Gas control	No
Top optics	Yes
Bottom optics	No
Dispenser	No
**Protocol parameters**	
Wavelengths ThT (nm)	Ex. 446<br>Em. 490
Excitation bandwidth (nm)	5
Dynamic range	Automatic
Measurement time (ms)	100

Table S2 – Plate reader instrumental parameters for the ThT binding assay experiments.

Figure S1 – Fluorescence microscope experimental setting scheme

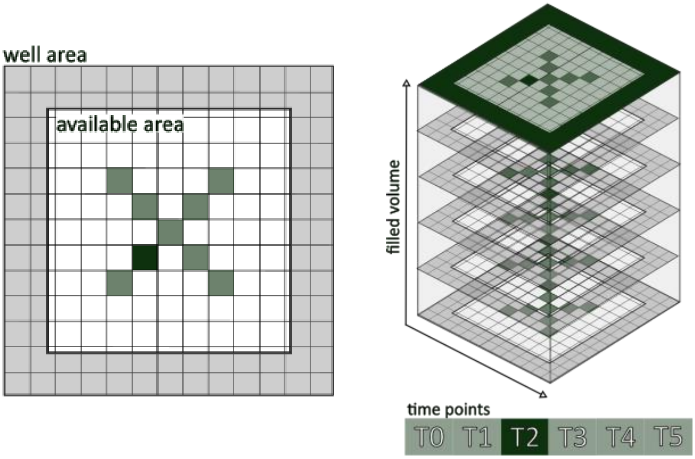

Figure S1-- Scheme of the system operation of the fluorescence microscope. The grid corresponds to the well areas and the square to slices of the well volume. Each selected plane from the well are divided in smaller areas (the square size and number depend on the magnification). In green are represented the areas selected for measurement, and darker green would represent the area selected for visualisation. Each green area is measured once per time point.

Figure S2 – WT-PD1 and WT-PD2 chemical structures

A

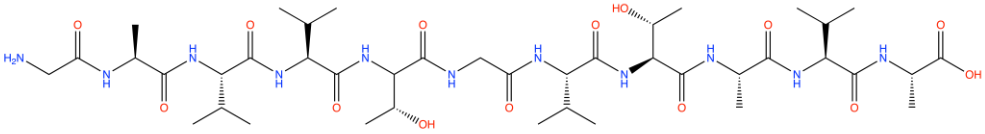

B

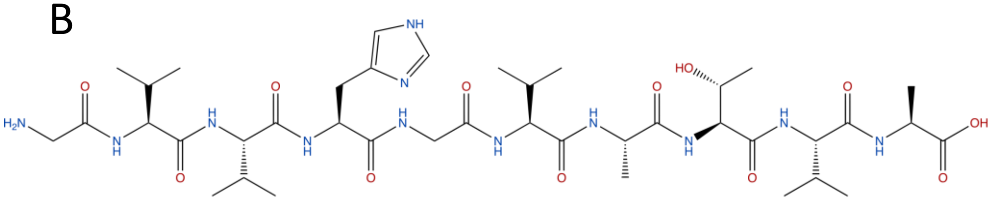

Figure S2 – Chemical structures of A) $_{68}$GAVVTGVTAVA$_{78}$ (WT-PD1) and B) $_{47}$GVVHGVATVA$_{56}$ (WT-PD2)

Figure S3 – Effect of sonication and ionic strength of AA buffer on ThT fluorescence binding assay of WT-PD1

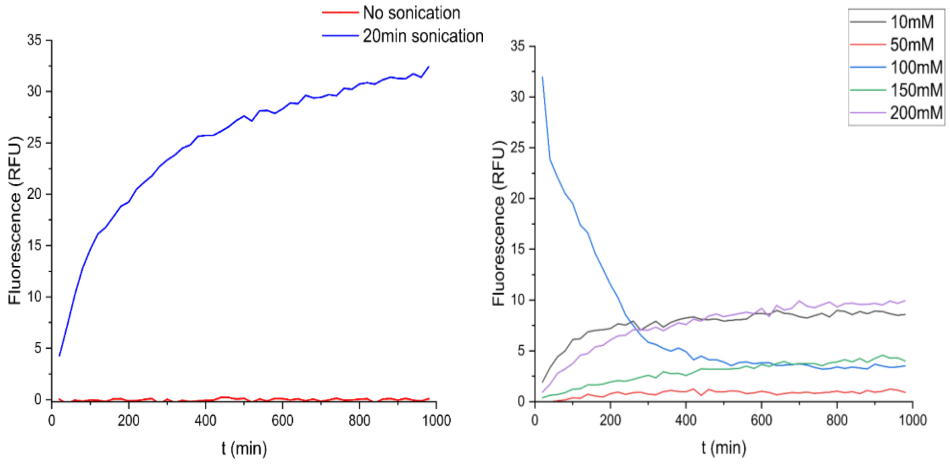

Figure S3 – ThT binding assay curves of WT-PD1 peptides from α-synuclein at a concentration of 100 µM peptide and ThT. Left – the fluorescence intensity was measured with the sample without sonication (red curve) and sonicated for 20 minutes (blue curve). Right – the fluorescence intensity was monitored for different values of ammonium acetate concentration of: 10 mM (black); 50 mM (red); 100 mM (blue); 150 mM (green) and 200 mm (purple). Note that the 100 mM sample shows unexpected behaviour, most likely resulting from experimental error or signal fluctuation under low-fluorescence conditions.

Figure S4 – Fluorescence microscopy images recorded without sonication at 5 minutes and 55 hours after sample preparation for WT-PD1 and WT-PD2

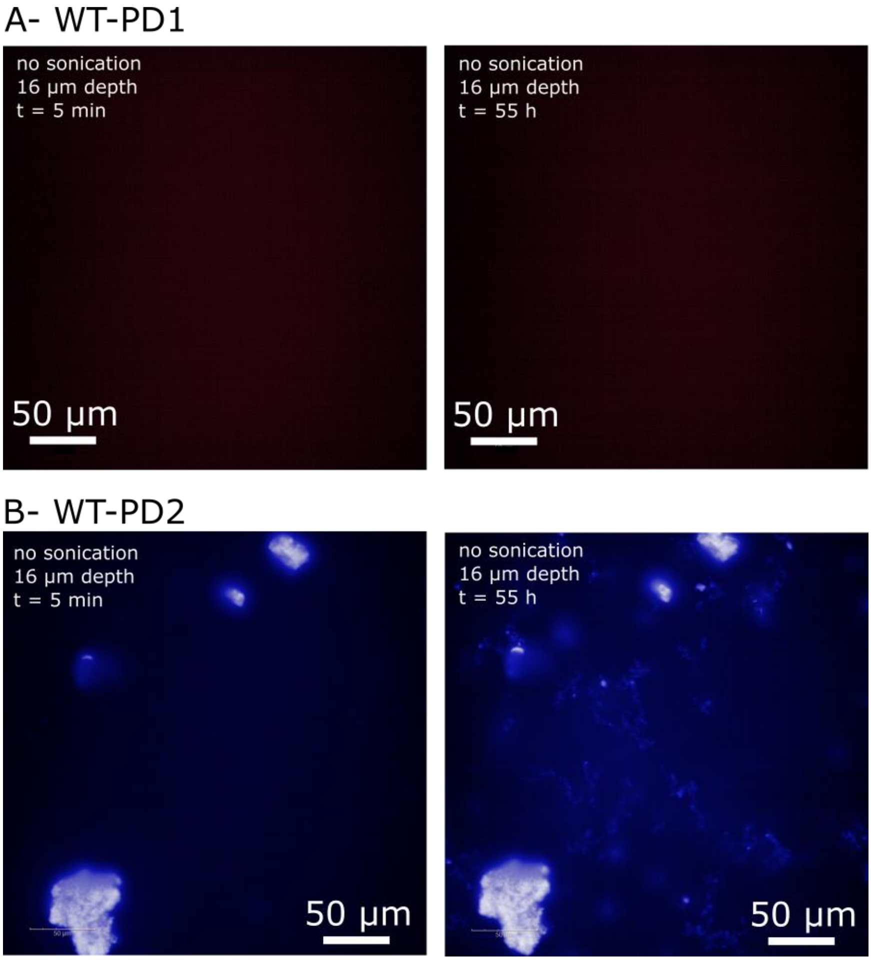

Figure S4 - ThT binding microscopy pictures of non-sonicated samples from A- WT-PD1 at 16 μm depth at t = 5 minutes and t = 55 hours after sample preparation. No fluorescence observed. And B- WT-PD2 at 16 μm depth at t = 5 minutes and t = 55 hours after sample preparation. Fluorescent features are observed from the start and are appearing weakly over time.

Figure S5 – Fluorescence microscopy images of WT-PD1 over time for two different well depths

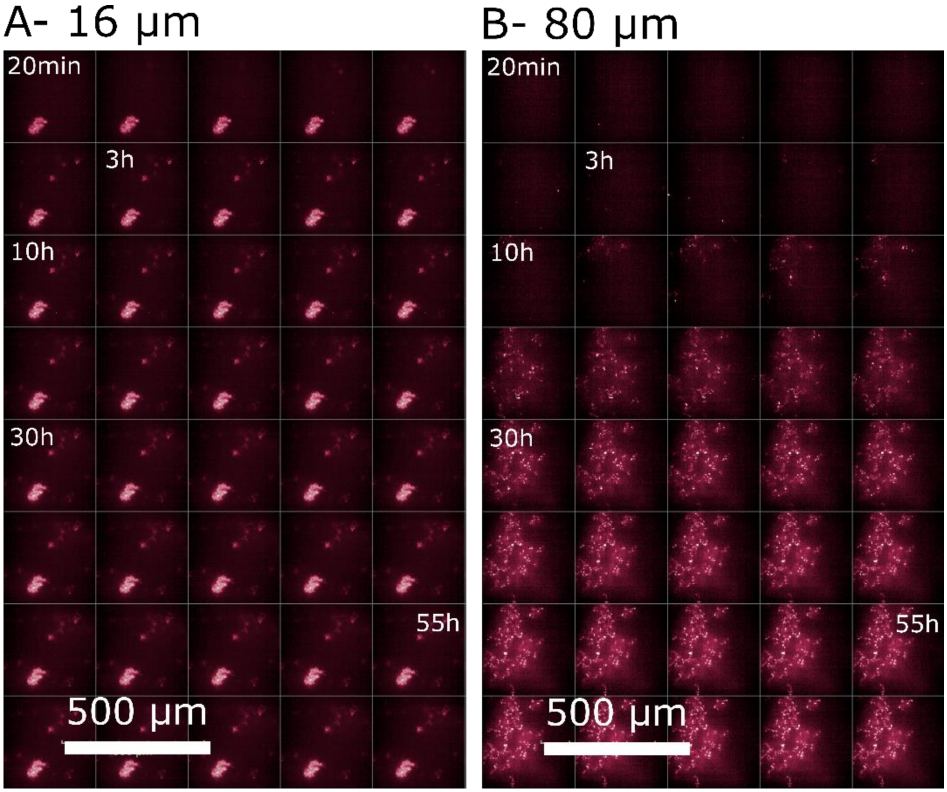

Figure S5 – ThT binding fluorescence microscopy pictures of WT-PD1 recorded over 68 hours, every 15 minutes from the end of sonication for the first 4 hours and then every hour for the remaining time. A- At 16 µm in the well and B- at 80 µm in the well. Pictures from Figure 3 in the main text are extracted from this timeline (only a portion of the pictures is displayed).

Figure S6 - Extracted MS spectra of WT-PD1 from each mobility peak present over time and corresponding MS zoomed on isotopic distribution

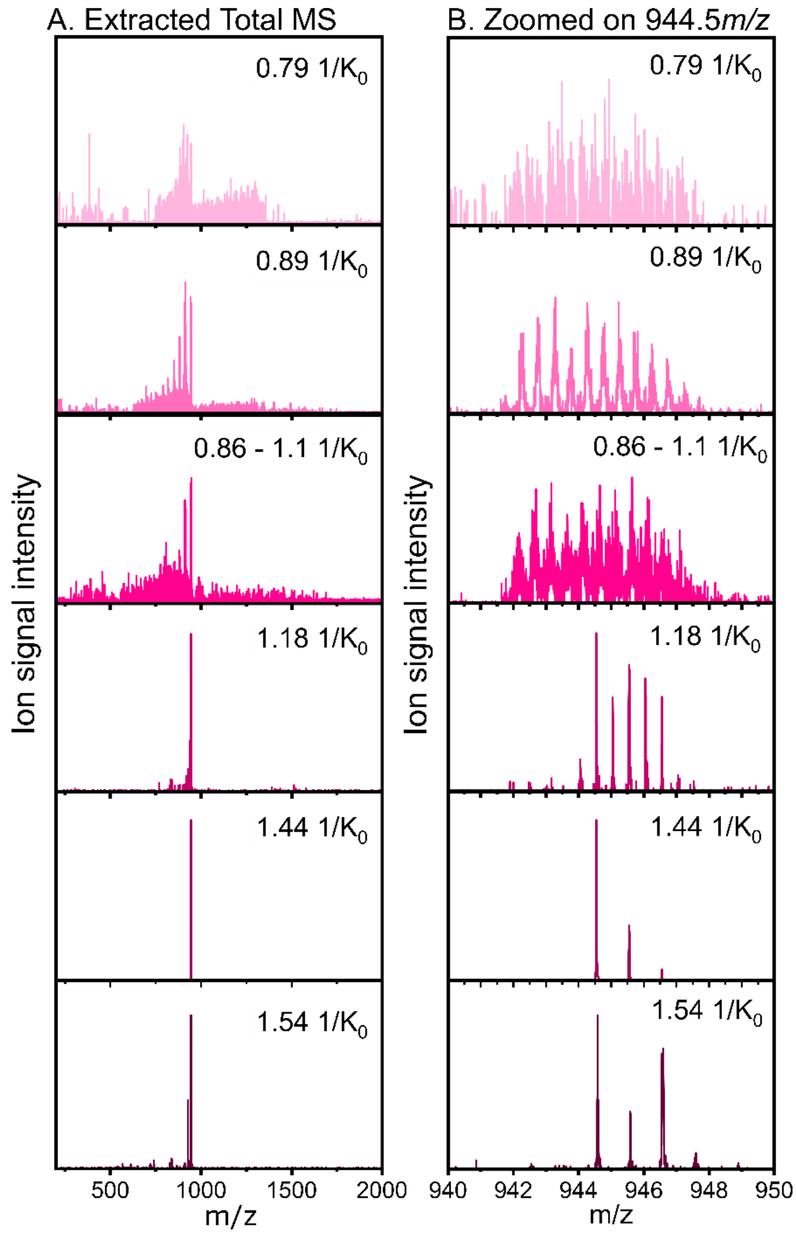

Figure S6 – Extracted MS from WT-PD1 from each ion mobility peak observed in Figure 4 of quadrupole selected 944.5 m/z. A- MS of the full recorded m/z range and B- MS zoomed on the isotopic distribution of 944.5 m/z

Figure S7 - Zoomed MS spectra of WT-PD1 at time points pre, peri and post-aggregation

The t_0, t_2 and t_5 MS corresponding to the mobilograms from Figure 2 in the main text are plotted,

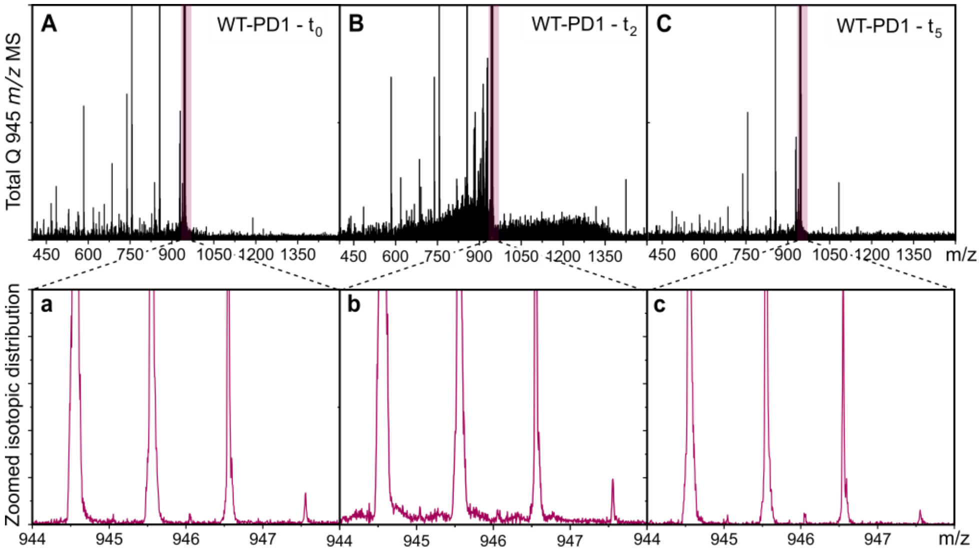

Figure S7 - A, B and C – Zoomed total quadrupole selected MS corresponding to time points t_0, t_2 and t_5 from WT-PD1 944.5 m/z. a, b and c – zoomed into the isotopic distribution of 944.5 m/z.

showing only small differences between time points. An increase of the baseline is observed at the time point where oligomers are present (t_2), i.e. under the 944.5 *m/z* area and under a mass range of higher mass to charge ratio from 1000 to 1400 *m/z.* This baseline increase is not observed at t_0 before aggregation or at t_5 after aggregation, when oligomers are not observed in the mobility spectra. This broad baseline increase is characteristic of oligomeric growth; however, it is relatively low when measured with the TIMS-Qq-ToF.[61] Additional peaks at lower m/z (e.g. < 944.5 m/z) are observed in each mass spectrum, which correspond to the fragmentation of ions (not necessarily oligomers). This is likely due to ion heating in the instrument occurring between the mobility cell and the mass analyser.[52] The isotopic distribution from the total MS does not allow to assert the presence of oligomers. The abundance of oligomers is overshadowed by the intense signal of the monomeric units.

Figure S8 – Replicates of the mobility over time measurements of the selected 944.5 *m/z* from WT-PD1

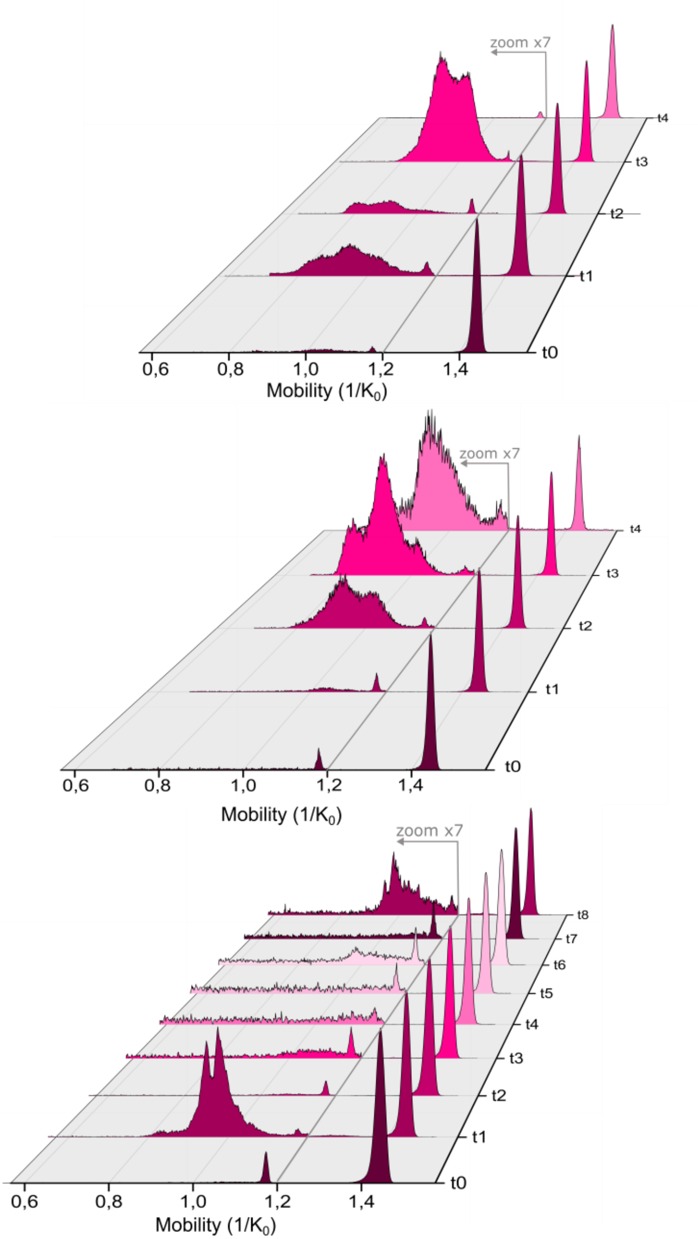

Figure S8 – Reduced ion mobility spectra of WT-PD1 mass selected 944.5 m/z plotted over time for three replicates of the experiment presented Figure 4. Intensity of the signal at values under 1.2 1/K_0 were zoomed seven times to optimise visibility.

Figure S9 – Fluorescence microscopy images of WT-PD2 over time for two different well depths

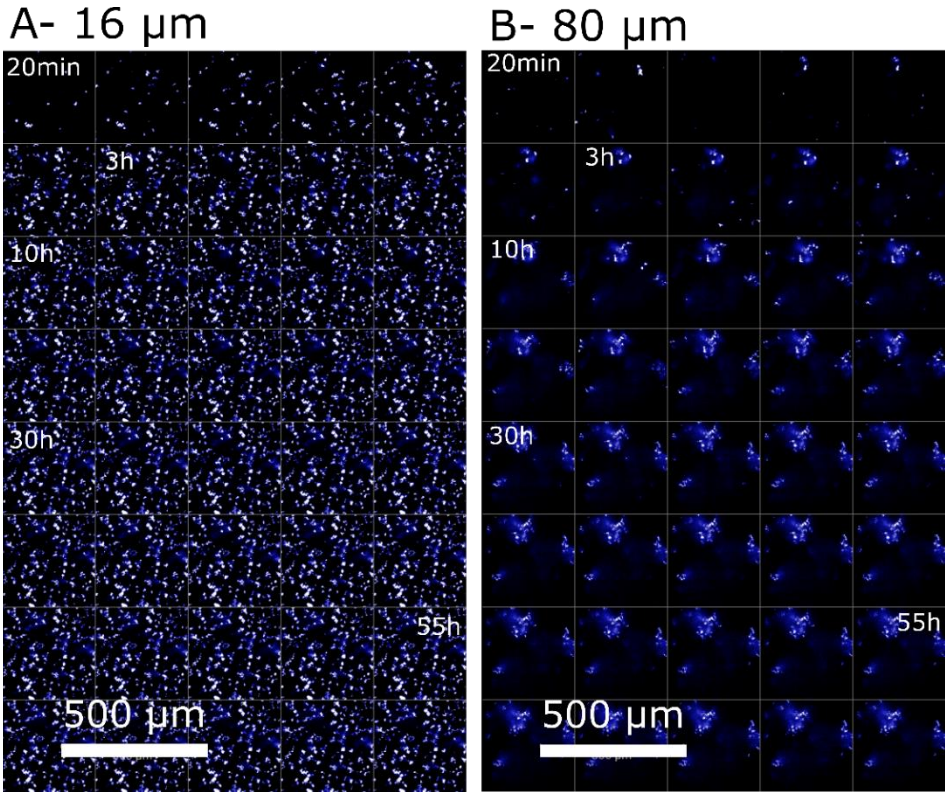

Figure S9 - ThT binding fluorescence microscopy pictures of WT-PD2 recorded over 68 hours, every 15 minutes from the end of sonication for the first 4 hours and then every hour for the remaining time. A- At 16 µm in the well and B- at 80 µm in the well. Pictures from Figure 3 in the main text are extracted from this timeline (only a portion of the pictures are displayed).

Figure S10 - Extracted MS spectra of WT-PD2 from each mobility peak present over time and corresponding MS zoomed on isotopic distribution

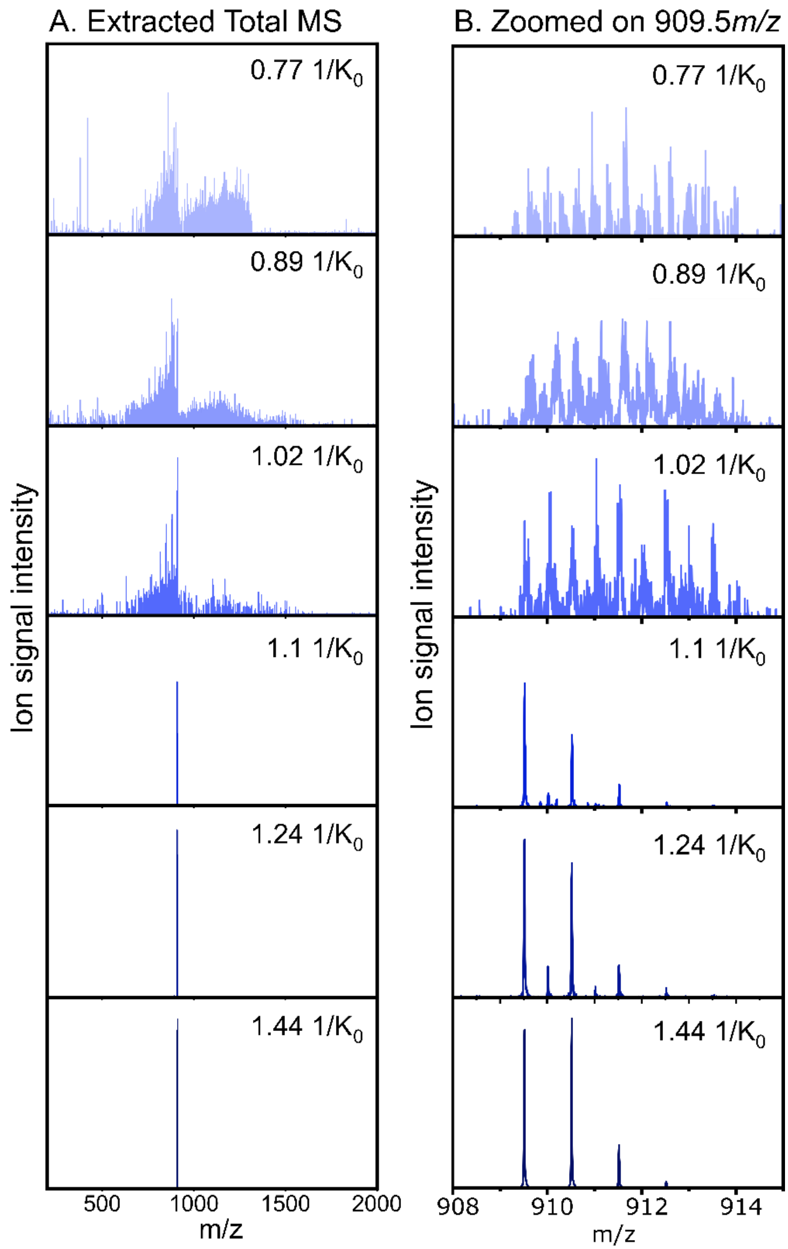

Figure S10 - Extracted MS from WT-PD2 from each ion mobility peak observed Figure 7 of quadrupole selected 909.5 m/z. A- MS of the full recorded m/z range and B- MS zoomed on the isotopic distribution of 909.5 m/z.

Figure S11 - Zoomed MS spectra of WT-PD2 at time points pre, peri and post-aggregation

The t_0, t_2, t_3 and t_6 MS corresponding to the mobilograms from Figure 4 in the main text are plotted.

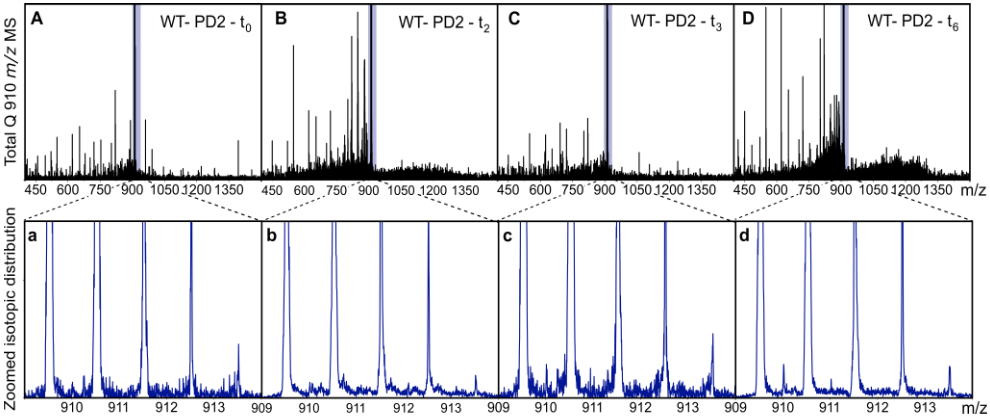

Figure S11 - A, B, C and D – Zoomed total quadrupole selected MS corresponding to time points t_0, t_2, t_3 and t_5 from WT-PD2 909.5 m/z. a,b, c and d – zoomed into the isotopic distribution of 909.5 m/z. t_0 and t_3 are time points when no higher order mobility peaks are observed, as opposed to t_2 and t_6.

At t_2 and t_6, only an increase of baseline under certain *m/z* is observed in addition to the typical fragments at *m/z* lower than 909.5 *m/z* due to ion heating. Figure S10.b and d, shows that when the isotopic distribution is examined in greater detail, it becomes evident that ions with a charge state greater than 2 are not resolved.

Figure S12 – Replicates of the mobility over time measurement of the selected 909.5 *m/z* from WT-PD2

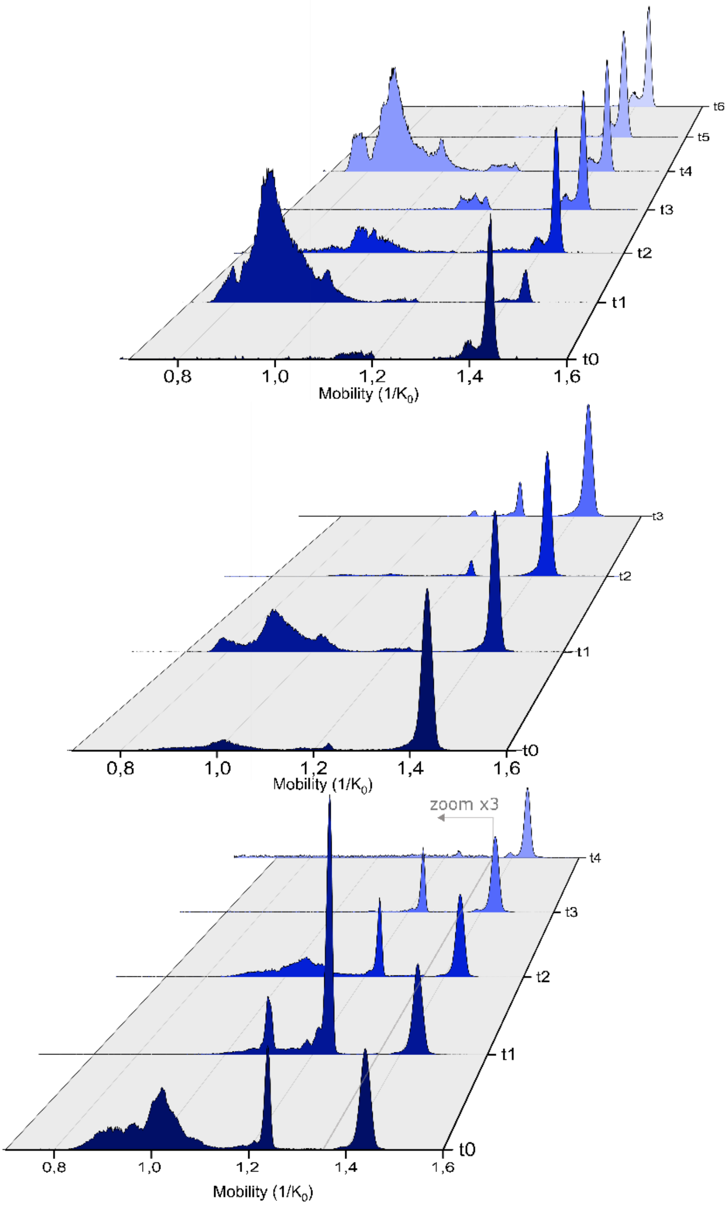

Figure S12 - Reduced ion mobility spectra of WT-PD2 mass selected 909.5 m/z plotted over time for three replicates of the experiment presented Figure 7. Bottom spectra - Intensity of the signal at values under 1.35 1/K_0 were zoomed seven times to optimise visibility.
